# New record and dietary ecology of a poorly known frog, *Amolopsshihaitaoi* Wang, Li, Du, Hou & Yu, 2022 (Amphibia, Anura, Ranidae), from Ha Giang Province, Vietnam

**DOI:** 10.3897/BDJ.11.e104316

**Published:** 2023-05-11

**Authors:** Sonephet Siliyavong, Ngoc Van Hoang, Tao Thien Nguyen, Truong Nguyen, Anh Van Pham

**Affiliations:** 1 Pakse Teacher Training College, 13 South Road, Champasak, Laos Pakse Teacher Training College, 13 South Road Champasak Laos; 2 Thai Nguyen University of Education, Thai Nguyen University, Thai Nguyen, Vietnam Thai Nguyen University of Education, Thai Nguyen University Thai Nguyen Vietnam; 3 Institute of Genome Research, Vietnam Academy of Science and Technology, Hanoi, Vietnam Institute of Genome Research, Vietnam Academy of Science and Technology Hanoi Vietnam; 4 Institute of Ecology and Biological Resources, Vietnam Academy of Science and Technology, Hanoi, Vietnam Institute of Ecology and Biological Resources, Vietnam Academy of Science and Technology Hanoi Vietnam; 5 Graduate University of Science and Technology, VAST, Hanoi, Vietnam Graduate University of Science and Technology, VAST Hanoi Vietnam; 6 Faculty of Environmental Sciences, University of Science, Vietnam National University, Ha Noi, 334 Nguyen Trai Road, Hanoi, Vietnam Faculty of Environmental Sciences, University of Science, Vietnam National University, Ha Noi, 334 Nguyen Trai Road Hanoi Vietnam; 7 The Center for Biodiversity & Environment Research, Tay Bac University, Son La City, Son La Province, Son La, Vietnam The Center for Biodiversity & Environment Research, Tay Bac University, Son La City, Son La Province Son La Vietnam

**Keywords:** distribution, new records, morphology, prey items, stomach contents

## Abstract

**Background:**

The Hekou Torrent Frog (*Amolopsshihaitaoi*) was recently discovered from southern China and northern Vietnam in 2022. The knowledge about natural history and feeding ecology of this species is virtually lacking.

**New information:**

Based on our recent fieldwork in northern Vietnam, we report a new population of *A.shihaitaoi* from Ha Giang Province. In this study, we provide novel data on the diet of *A.shihaitaoi*, based on stomach content analyses of 36 individuals (17 males and 19 females). A total of 36 prey categories with 529 items, comprising 515 items of invertebrates and 14 unidentified items, were found in the stomachs of *A.shihaitaoi*. The dominant prey items of the species were Hymenoptera (Formicidae), Orthoptera (Acrididae), Lepidoptera (Lepidoptera other), Mantodea (Mantidae) and Araneae. The importance index (Ix) of prey categories ranged from 7.1% to 11.5%. Hymenoptera (Formicidae) had the highest frequency of prey items, found in 36 stomachs.

## Introduction

Studying dietary ecology is crucial for understanding natural history, population fluctuations and the impact of habitat change on frog populations (Ogoanah and Uchedike 2011). Identifying prey taxa for each species will help clarify the impact of frogs on local invertebrate fauna and determine which prey species are dietary resources for the frogs (Nakamura and Tominaga 2021). Some species have highly varied diets, but concentrate their consumption on a few prey categories (Siqueira et al. 2006, Lima et al. 2010, Pham et al. 2019), while others have a narrow or specialized diet on certain prey categories (Rödel and Braun 1999, Hirai and Matsui 2000, Solé et al. 2002, Pham et al. 2022). In addition, the diet of amphibian species depends on prey availability in the environment (Toft 1980, Donnelly 1991).

Diet differences between sexes may occur due to differences in energy expenditure and behaviour (Donnelly 1991, Valderrama-Vernaza et al. 2009) or in response to seasonal variations in prey availability (Maneyro et al. 2004). However, both males and females are usually capable of consuming prey of different sizes, so dietary differences between sexes generally occur in the number of prey consumed (Donnelly 1991, Valderrama-Vernaza et al. 2009) or in dietary composition (Brasileiro et al. 2010).

The genus *Amolops* Cope, 1865 currently contains 74 predominantly diurnal species that inhabit forest streams (Patel et al. 2021, Frost 2023). Despite a large number of species, dietary studies in the genus have only been done on *Amolopslarutensis* (Berry 2009). In Vietnam, studies on the diet have also been conducted on several amphibian species, including *Quasipaaverrucospinosa* in Thua Thien Hue Province (Ngo et al. 2014) and *Microhylabutleri*, *M.heymonsi* and *Odorranachapaensis* in Son La Province (Pham et al. 2019, Pham et al. 2022). Those studies demonstrated that these frogs have varied diets, with the majority being ants, beetles, dipterans and insect larvae.

The Hekou Torrent Frog (*Amolopsshihaitaoi*) was originally described from southern China (Yunnan and Guangxi Provinces) and northern Vietnam (Vinh Phuc, Cao Bang and Lao Cai Provinces) by Wang et al. (2022). In this study, we report the first record of *Amolopsshihaiaoi* from Ha Giang Province and provide the novel data on dietary ecology.

## Materials and methods

**Figure 1.**
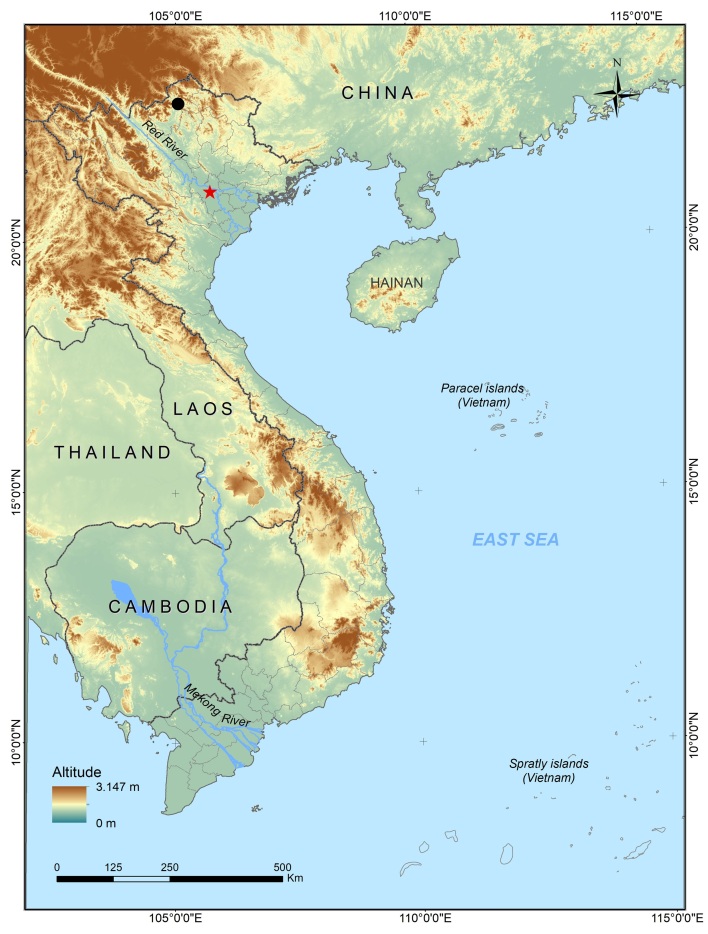
Map showing the survey sites in Ha Giang Province, northern Vietnam.

A field survey was conducted in Lung Vai Village, Phuong Do Commune, within Tay Con Linh Nature Reserve, Ha Giang Province, northern Vietnam (Fig. 1) by Ngoc Van Hoang and Sonphet Silyavong in August 2022. Frogs were collected by hand between 8:00 and 23:00 hrs following the guidelines approved by the American Society of Ichthyologists and Herpetologists for animal care (Beaupre et al. 2004). We used a stomach-flushing technique to obtain stomach contents without sacrificing them (Griffiths 1986, Leclerc and Courtois 1993, Solé et al. 2005). Prey items were preserved in 70% ethanol. Frogs were subsequently released at the collecting site after taking measurements of snout-vent length (SVL) and mouth width (MW) with a digital caliper to the nearest 0.01 mm.

For taxonomic identification, four individuals were collected for voucher specimens. After being photographed in life, these animals were anaesthetized and euthanized in a closed vessel with a piece of cotton wool containing ethyl acetate (Simmons 2002), fixed in 85% ethanol and subsequently stored in 70% ethanol. Specimens were subsequently deposited in the collection of the Thai Nguyen University of Education (TNUE), Thai Nguyen Province, Vietnam.

### Morphological characters

All measurements were taken with a caliper to the nearest 0.1 mm following Wang et al. (2022) and abbreviations are as follows: SVL: snout-vent length; HL: head length, from tip of snout to rear of jaws; HW: maximum head width, at the angle of jaws; SL: distance from anterior corner of eye to tip of snout; ED: eye diameter, from anterior corner to posterior corner of eye; DNE: distance from anterior corner of eye to posterior edge of nostril; IND: internarial distance; IOD: minimum distance between upper eyelids; UEW: maximum width of upper eyelid; TD: maximum tympanum diameter; FHL: forearm and hand length,from elbow to tip of third finger; TL: tibia length, from knee to heel; FL: foot length, from proximal end of inner metatarsal tubercle to tip of fourth toe; and TFL: length of foot and tarsus, from tibiotarsal joint to tip of fourth toe.

### Stomach content analysis

Prey items were identified using a microscope (Olympus SZ 700) and taxonomic identification keys (i.e. Naumann et al. (1991), Thai (2003), Johnson and Triplehorn (2005), Brusca et al. (2016)). The maximum length (L) and width (W) of each prey item were measured to the nearest 0.1 mm using either a caliper or a calibrated ocular micrometer fitted to a microscope. The volume (V) of prey item was calculated using the formula for a prolate spheroid (π = 3.14, Magnusson et al. (2003)): V=(4π/3)×(L/2)×(W/2)^2^ (mm³). The index of relative importance (IRI) was used to determine the importance of each food category. This index provides a more informed estimation of prey item consumption than any of the three components alone, using the following formula: IRI = (%F + %N + %V)/3 (Caldart et al. 2012), where F is the frequency of prey occurrence in stomachs and N is the total number of prey items concerning all prey items. We used the reciprocal Simpson’s heterogeneity index 1/D to calculate dietary heterogeneity: D = –∑[ni(ni–1)]/([N(N–1)]), where ni is the number of prey items in the i^th^ taxon category and N is the total number of prey items (Krebs 1999).

To estimate prey evenness, we used Shannon’s Index of Evenness. Evenness is calculated from the equation: J′ = H′/Hmax = H′/lnS. Hmax is the maximum diversity that could occur if all taxa had equal abundance. H′ = Hmax = lnS, S is the total number of prey taxa and H' is the Shannon-Weiner index of taxon diversity, calculated from the equation: H′ = –∑(Pi×lnPi), where Pi is the proportion of total prey items belonging to the taxon for the total prey items of the sample (Magurran 2004, Muñoz-Pedreros and Merino 2014).

Statistic analyses were performed using SPSS 20.0 software (SPSS Inc. Chicago, Illinois, USA), with the significance level set to P < 0.05 for all analyses. Data are presented as mean ± standard deviation (SD) unless otherwise noted. We used Kendall’s tau b statistics to examine the number of prey items and prey volume from frogs of different sexes. We used one-way analysis of variance (ANOVA) to examine the size of prey items collected between sexes.

## Data resources

For taxonomic identification, four individuals were collected as morphological analysis. In addition, a total of 40 adult individuals (20 males and 20 females) of *A.shihaitaoi* were collected from Ha Giang Province for stomach flushing.

## Taxon treatments

### Amolops shihaitaoi

Wang, Li, Du, Hou & Yu, 2022

96AB542A-0E70-5F73-BC46-014043073680

https://amphibiansoftheworld.amnh.org/Amphibia/Anura/Ranidae/Amolops/Amolops-shihaitaoi

#### Materials

- **Type status:** Other material. **Occurrence:** catalogNumber: LV 53; individualCount: 1; sex: male; lifeStage: adult; occurrenceID: F7C18AED-1D84-568E-ACB1-E231A190C353; **Taxon:** scientificNameID: Amolopsshihaitaoi; scientificName: *Amolopsshihaitaoi*; class: Amphibia; order: Anura; family: Ranidae; genus: Amolops; specificEpithet: shihaitaoi; scientificNameAuthorship: Wang, Li, Du, Hou & Yu, 2022; **Location:** country: Vietnam; countryCode: VN; stateProvince: Ha Giang; county: Ha Giang; municipality: Phuong Do; locality: near Lung Vai Village; verbatimElevation: 850 m; verbatimLatitude: 22°49'50''N; verbatimLongitude: 104°53'51''E; verbatimCoordinateSystem: WGS84; **Event:** eventDate: August 08; eventTime: 2022; eventRemarks: collected by N.V. Hoang and S. Silyavong; **Record Level:** language: en; collectionCode: Amphibians; basisOfRecord: PreservedSpecimen
- **Type status:** Other material. **Occurrence:** catalogNumber: LV 57; individualCount: 1; sex: male; lifeStage: adult; occurrenceID: 19DB3F80-2BF4-506C-9B98-59A11463C6B3; **Taxon:** scientificNameID: Amolopsshihaitaoi; scientificName: *Amolopsshihaitaoi*; class: Amphibia; order: Anura; family: Ranidae; genus: Amolops; specificEpithet: shihaitaoi; scientificNameAuthorship: Wang, Li, Du, Hou & Yu, 2023; **Location:** country: Vietnam; countryCode: VN; stateProvince: Ha Giang; county: Ha Giang; municipality: Phuong Do; locality: near Lung Vai Village; verbatimElevation: 850 m; verbatimLatitude: 22°49'50''N; verbatimLongitude: 104°53'51''E; verbatimCoordinateSystem: WGS84; **Event:** eventDate: August 08; eventTime: 2022; eventRemarks: collected by N.V. Hoang and S. Silyavong; **Record Level:** language: en; collectionCode: Amphibians; basisOfRecord: PreservedSpecimen
- **Type status:** Other material. **Occurrence:** catalogNumber: LV 34; individualCount: 1; sex: female; lifeStage: adult; occurrenceID: 417C2CA5-B977-522E-A43C-242460636B42; **Taxon:** scientificNameID: Amolopsshihaitaoi; scientificName: *Amolopsshihaitaoi*; class: Amphibia; order: Anura; family: Ranidae; genus: Amolops; specificEpithet: shihaitaoi; scientificNameAuthorship: Wang, Li, Du, Hou & Yu, 2024; **Location:** country: Vietnam; countryCode: VN; stateProvince: Ha Giang; county: Ha Giang; municipality: Phuong Do; locality: near Lung Vai Village; verbatimElevation: 850 m; verbatimLatitude: 22°49'50''N; verbatimLongitude: 104°53'51''E; verbatimCoordinateSystem: WGS84; **Event:** eventDate: August 08; eventTime: 2022; eventRemarks: collected by N.V. Hoang and S. Silyavong; **Record Level:** language: en; collectionCode: Amphibians; basisOfRecord: PreservedSpecimen
- **Type status:** Other material. **Occurrence:** catalogNumber: LV 59; individualCount: 1; sex: female; lifeStage: adult; occurrenceID: A1C87F5B-3270-5C69-9760-39D16CB01387; **Taxon:** scientificNameID: Amolopsshihaitaoi; scientificName: *Amolopsshihaitaoi*; class: Amphibia; order: Anura; family: Ranidae; genus: Amolops; specificEpithet: shihaitaoi; scientificNameAuthorship: Wang, Li, Du, Hou & Yu, 2025; **Location:** country: Vietnam; countryCode: VN; stateProvince: Ha Giang; county: Ha Giang; municipality: Phuong Do; locality: near Lung Vai Village; verbatimElevation: 850 m; verbatimLatitude: 22°49'50''N; verbatimLongitude: 104°53'51''E; verbatimCoordinateSystem: WGS84; **Event:** eventDate: August 08; eventTime: 2022; eventRemarks: collected by N.V. Hoang and S. Silyavong; **Record Level:** language: en; collectionCode: Amphibians; basisOfRecord: PreservedSpecimen

#### Description

Morphological characteristics of the specimens from Ha Giang Province, Vietnam, agreed with the description of Wang et al. (2022): SVL 33.2–33.6 mm in males (n = 2), 42.2–43.8 mm in females (n = 2); head wider than long; snout short, round; nostril lateral, wider than interorbital distance and upper eyelid width; tympanum smaller than half eye diameter; vomerine teeth present; vocal openings absent in males. Forelimb robust; relative finger lengths I < II < IV < III; fingers free of webbing; tips of fingers expanded into discs; circummarginal groove on disc of the first finger present; palmar tubercles two, oval; nuptial pads present in males. Hind-limb long, thigh shorter than tibia; toes fully webbed, tips of toes expanded into discs; inner metatarsal tubercle distinct; tarsal fold and tarsal glands absent; tibiotarsal articulation reaching to snout when limb adpressed along body (see measurements in Table 1). The specimens from Vietnam slightly differ from the type series from China in having spines on nuptial pads not clearly distinct in males and this may be due to the difference in sampling time (in August in Vietnam and in June in China).

Skin. Dorsal surface rough and granular with denser small translucent, dorsolateral folds absent; temporal and loreal region with small white spines; supratympanic fold present; ventral smooth.

**Figure 2.**
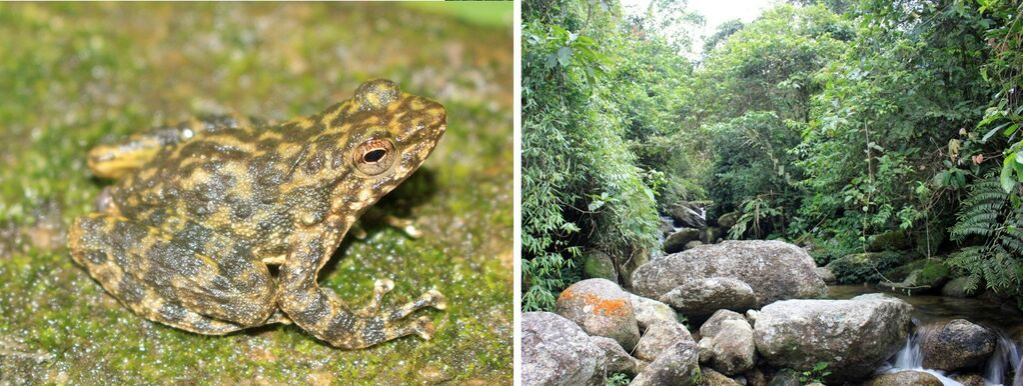
Adult male (Left) and natural habitat (Right) and of *Amolopsshihaitaoi* in Ha Giang Province, northern Vietnam.

Colouration in life. Dorsal surface olive-brown with dark brown patches and dark irregular transverse bars on limbs; flanks olive-brown with warts dark or white; ventral surface white, ventral surface of limbs cream (Fig. 2).

#### Distribution

In Vietnam, this species was previously recorded from Lao Cai, Cao Bang and Vinh Phuc Provinces (Wang et al. 2022). This is the first record of the species in Ha Giang Province. Elsewhere, this species is known from southern China (Wang et al. 2022).

#### Ecology

Specimens of *A.shihaitaoi* were found on the cliff of waterfalls and large rocks in the streams between 20:00 and 23:00 h. The surrounding habitat was evergreen forest of large hardwood and shrub (Fig. 2).

#### Diet

A total of 40 adult individuals (20 males and 20 females) of *A.shihaitaoi* were collected from Ha Giang Province for stomach flushing, of which three frogs (or 7.5%) had empty stomachs. We identified 529 prey items, including 515 items of animals and 14 unidentified items. Males had 190 prey items, while females had 339 prey items.

The number of prey items per individual ranged 2–40 items (average 14.69 ± 9.19 items). The number of prey items in males ranged 2–40 (average 11.18 ± 8.41 items), while in females, it ranged 4–35 (average 17.84 ± 8.90 items) (Kendall’s tau b: tau = 0.355, P = 0.004) (Table 2).

Mean prey item length was 5.43 ± 3.99 mm (ranging from 1.00 to 40.00 mm) and mean prey item width was 1.64 ± 1.39 mm (ranging from 1.00 to 40.00 mm) in both sexes.

Mean prey item length in males was 4.48 ± 3.30 mm (ranging from 1.00 to 30.00 mm) and ranged from 1.00 to 40.00 mm in females (average 5.95 ± 4.24 mm); those were significantly different from each other (*F*_1,528_ = 1.449, *P* = 0.018) as well as mean prey item width in males being 1.60 ± 1.69 mm (ranging from 0.4 to 10.00 mm) and ranging from 0.40 to 7.00 mm in females (average 1.66 ± 1.19 mm); those were significantly different from each other (*F*_1,413_ = 2.018, *P* = 0.001).

The average volume per individual was 242.73 ± 248.19 mm^3^ (ranging from 10.71 to 848.06 mm^3^). In which, the average volume per male individual was 140.57 ± 210.38 mm^3^ (ranging from 10.71 to 688.21 mm^3^) and 334.16 ± 248.55 mm^3^ (ranging from 36.85 to 848.06 mm^3^) in female; those were significantly different from each other (tau = 0.472, *P* < 0.01) (Table 2).

**Figure 3.**
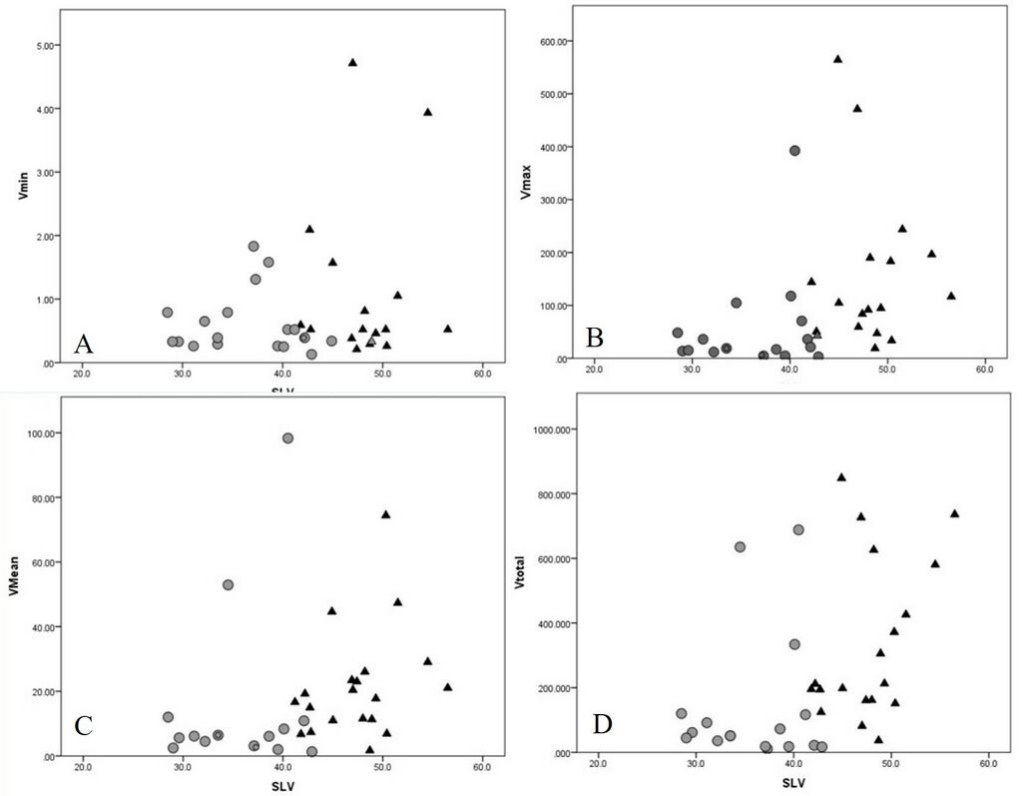
Relationships between the frog SVL and the minimum (A), maximum (B) and the mean (C) prey item volume and the total prey volume (D). Dots: Males; Open triangles: Females; Vmin = minimum prey item volume (mm^3^); Vmax = maximum prey item volume (mm^3^); Vmean = mean prey item volume (mm^3^); Vtotal = the total prey volume (mm^3^).

There was not a positive correlation between the frog SVL and the minimum prey volume (tau = 0.47, P = 0.672) (Fig. 3 A), while there were correlations between the frog SVL and the maximum prey item volume (tau = 0.356, P < 0.01), mean prey item volume (tau = 0.354, P = 0.01) and the total prey volume (tau = 0.351, P < 0.01) (Fig. 3 B-D).

We identified 35 different categories of prey and other unidentified subjects in the stomachs of *A.shihaitaoi* with insects being the main food component, including 11 orders and other invertebrate groups, namely Opiliones, Araneae, Crustacea and Diplopoda (Table 3).

The most commonly consumed prey items were Formicidae (15.12%), followed by Acrididae (13.42%), Mantidae (9.64%), Araneae (9.45%) and other Lepidoptera (8.70%). While the most frequently foraged prey group was Lepidoptera other (15.28%), followed by Formicidae (14.58%), Acrididae (11.11%), Araneae (8.33%) and Mantidae (4.17%). In the comparisons by the IRI, Formicidae (11.5%), Acrididae (11.0%), other Lepidoptera (8.5%), Araneae (8.0%) and Mantidae (7.1%) were identified as the most important prey groups (Table 3).

The total dietary breadth of *A.shihaitaoi* from Vietnam was 13.22 (Simpson’s index of diversity) and Shannon’s evenness was 0.82. Adult females (19 prey categories) consumed more diverse prey than adult males (16 prey categories). The diversity index of prey categories of adult males (11.11 with an evenness index of 0.41) was also lower than that of adult females (11.48 with an evenness index of 0.61) (Table 4).

There was an overlap of more than 65% in the diet of males and females. The trophic spectrum of males consisted of 24 prey categories, the most important groups (with IRI > 6) being Araneae, Lepidoptera, Blattidae, Hepialidae, Formicidae and Acrididae, while the trophic spectrum of females comprised 26 prey categories, with Formicidae, Acrididae, Mantidae, Gryllotalpidae and Coleoptera being the most important prey categories.

**Figure 4.**
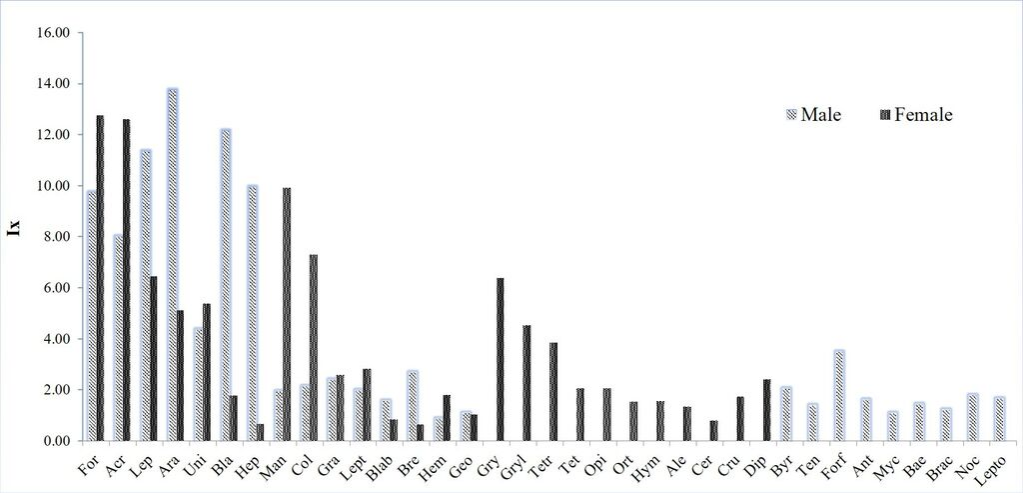
Importance indices (Ix) for prey categories consumed by males (cross) vs. females (black) of *Amolopsshihaitaoi* in Vietnam. For: Formicidae, Acr: Acrididae, Lep: Lepidoptera other, Ara: Araneae, Uni: Unidentified, Bla: Blattidae, Hep: Hepialidae, Man: Mantidae, Col: Coleoptera other, Gra: Gracillariidae, Lept: Leptophlebiidae, Blab: Blaberidae, Bre: Brentidae, Hem: Hemiptera other, Geo: Geometridae, Gry: Gryllotalpidae, Gryl: Gryllidae, Tetr: Tetrigidae, Tet: Tettigoniidae, Opi: Opiliones, Ort: Orthoptera other, Hym: Hymenoptera other, Ale: Aleyrodidae, Cer: Cercopidae, Cru: Crustacea, Dip: Diplopoda, Byr: Byrrhidae, Ten: Tenebrionidae, Forf: Forficulidae, Ant: Anthomyiidae, Myc: Mycetophilidae, Bae: Baetidae, Brac: Braconidae, Noc: Noctuidae, Lepto: Leptoceridae.

Byrrhidae, Tenebrionidae, Forficulidae, Anthomyiidae, Mycetophilidae, Baetidae, Braconidae, Noctuidae and Leptoceridae were found exclusively in the diet of males, whereas Opiliones, Crustacea, Diplopoda, other Hymenoptera, Gryllotalpidae, Gryllidae, Tetrigidae, Aleyrodidae, Cercopidae, Tettigoniidae and Orthoptera were found only in the diet of females. Despite these differences, Formicidae and Acrididae were identified as the most important prey categories for both males and females (Fig. 4).

## Discussion

Most studies show that insects are the main diet of frogs, which is also the most diverse prey group (Werner et al. 1995, Hothem et al. 2009, Brito et al. 2013, Ngo et al. 2014, Pham et al. 2019, Pham et al. 2022). Anurans are often feeding on spiders, beetles, grasshoppers, cockroaches, termites and ants (e.g. Biavati et al. (2004), Laufer (2004), Caldart et al. (2012), Pham et al. (2019), Pham et al. (2022)).

Our results showed that *A.shihaitaoi* preys on a wide variety of insects, similar to other studies on the diet of frogs from Vietnam (Ngo et al. 2014, Pham et al. 2019, Pham et al. 2022). The most common prey items of *A.shihaitaoi* were beetles, chalk wings, crickets, grasshoppers, ants and other groups, these being similar to the diet of many frogs (Ngo et al. 2014, Pham et al. 2019, Pham et al. 2022). These are terrestrial prey, which is in line with their general habitat use (Pham et al. 2019). Besides prey categorized as insects, *A.shihaitaoi* also consumed other invertebrates, viz. spiders, earwigs and crabs.

We also found differences in the dietary composition between males and females of *A.shihaitaoi*. While 26 prey catergories were recorded in females, only 24 were recorded in the males. These differences may be related to behavioural differences, as females do not defend calling sites or engage in agonistic interactions, allowing them to feed more frequently (Brasileiro et al. 2010, Caldart et al. 2012). Despite a varied diet, *A.shihaitaoi* had a narrow niche breadth with a few categories comprising most of the diet (frequency), including Lepidoptera (20.83%), Hymenoptera (16.67%), Orthoptera (15.97%) and Coleoptera (9.72%) (Table 2). Our estimation of prey availability suggested that food resources for *A.shihaitaoi* were abundant in the studied streams, allowing the co-existence of both adult males and females, despite their high dietary overlap of > 65% between males and females.

As females have a larger body size than males, they are more likely to consume larger prey items than males (Le et al. 2020). In this study, we found also the prey volume of *A.shihaitaoi* in females was greater than that in males. This is consistent with the scale-efficiency hypothesis (Forsman 1996).

## Supplementary Material

XML Treatment for Amolops shihaitaoi

## Figures and Tables

**Table 1.** Measurements (in mm) of *Amolopsshihaitaoi* collected from Ha Giang Province, Vietnam (M: male; F: female)

	**LV 53**	**LV57**	**LV34**	**LV59**
Sex	M	M	F	F
SVL	33.6	33.2	42.2	43.8
HL	11.2	10.8	14.0	14.1
HW	12.9	12.4	15.5	15.8
SL	4.8	4.4	5.4	5.6
IND	5.0	4.6	5.7	5.8
IOD	2.9	3	3.5	3.6
UEW	3.1	3.2	4.0	3.8
ED	4.8	4.8	5.6	5.9
TD	1.4	1.5	1.4	1.6
DNE	2.1	1.9	2.5	2.6
FHL	18.5	17.6	20.0	20.5
TL	20.5	19.6	23.3	25.1
TFL	27.5	27.3	31.2	32.2
FL	18.2	17.7	21.1	21.8

**Table 2.** Summary (Total, Mean, SD and range) of the prey item number (N), width (W), length (L) and volume (V) data for males and females (in mm for W and L; in mm^3^ for V).

	**Prey item**			
	**Male (n = 190)**		**Female (n = 339)**	
W	0.40–10.00	1.60 ± 1.69	0.40–7.00	1.66 ± 1.19
L	1.00–30.00	4.48 ± 3.30	1.00–40.00	5.95 ± 4.24
V_total	10.71–688.21	140.57 ± 210.38	36.85–848.06	334.16 ± 248.55
V_minimum	0.13–1.83	0.62 ± 0.5	0.21–4.71	1.03 ± 1.26
V_maximum	2.88–392.5	53.2 ± 94.03	18.84–564.15	145.84 ± 146.43
V_mean	1.32–98.32	14.46 ± 24.7	1.67–74.42	21.98 ± 17.46
N	2.00–40.00	11.18 ± 8.41	4.00–35.00	17.84 ± 8.90

**Table 3.** Dietary composition (%) of *Amolopsshihaitaoi* with regards to frequency of occurrence, numeric proportion, volume proportion and overall importance index of each prey category (n = 529 prey items).

Prey category	Frequency	Numeric proportion	Volume proportion	Importance index
**Opiliones**	0.69	2.84	0.36	1.30
**Araneae**	8.33	9.45	6.15	**7.98**
**Crustacea**	1.39	0.38	1.46	1.08
**Diplopoda**	2.08	1.32	0.92	1.44
**Blattodea**				
Blaberidae	1.39	0.95	1.00	1.11
Blattidae	3.47	3.59	7.52	4.86
**Coleoptera**				
Brentidae	2.08	1.51	0.56	1.39
Byrrhidae	0.69	0.76	0.71	0.72
Tenebrionidae	1.39	0.38	0.04	0.60
Coleoptera other	4.86	2.84	9.67	5.79
**Dermaptera**				
Forficulidae	0.69	2.84	0.32	1.28
**Diptera**				
Anthomyiidae	1.39	0.38	0.23	0.66
Mycetophilidae	0.69	0.57	0.07	0.44
**Ephemeroptera**				
Baetidae	0.69	0.57	0.35	0.54
Leptophlebiidae	2.08	2.46	3.48	2.67
**Hemiptera**				
Aleyrodidae	0.69	1.51	0.28	0.83
Cercopidae	0.69	0.57	0.13	0.46
Hemiptera other	1.39	0.57	2.82	1.59
**Hymenoptera**				
Braconidae	0.69	0.38	0.31	0.46
Formicidae	14.58	15.12	4.86	**11.52**
Hymenoptera other	1.39	0.38	1.10	0.96
**Lepidoptera**				
Geometridae	1.39	0.38	1.44	1.07
Gracillariidae	2.08	1.13	4.78	2.67
Hepialidae	1.39	0.57	7.77	3.24
Noctuidae	0.69	0.95	0.35	0.66
Lepidoptera other	15.28	8.70	1.61	**8.53**
**Mantodea**				
Mantidae	4.17	9.64	7.55	7.12
**Orthoptera**				
Acrididae	11.11	13.42	8.59	**11.04**
Gryllotalpidae	0.69	4.35	8.03	4.36
Gryllidae	0.69	3.59	4.83	3.04
Tetrigidae	2.08	3.21	1.92	2.40
Tettigoniidae	0.69	1.32	2.05	1.36
Orthoptera other	0.69	0.19	2.17	1.02
**Trichoptera**				
Leptoceridae	1.39	0.57	0.11	0.69
**Unidentified**	6.25	2.65	6.47	5.12

**Table 4.** Simpson’s Index of Diversity and Shannon’s Evenness between sexes in the diet of *Amolopsshihaitaoi* from Ha Giang Province, Vietnam.

**Sex**	**Simpson’s index 1/*D***	**Shannon’s evenness *E***
Males	11.11	0.41
Females	11.48	0.61
Total dietary	13.22	0.82
